# Influence of Organic Reinforcements on the Structural and Mechanical Properties of Epoxy Dental Composites

**DOI:** 10.7759/cureus.112378

**Published:** 2026-07-09

**Authors:** Indranil Lahiri, Partha Roy, Amandeep Jindal, Ajay Chhabra, Sayam Bhattacharya, Abhishek Sarkar, Vandana Chabbra, Priyanka Yadav

**Affiliations:** 1 Metallurgical and Materials Engineering, Indian Institute of Technology Roorkee, Roorkee, IND; 2 Bioscience and Bioengineering, Indian Institute of Technology Roorkee, Roorkee, IND; 3 Chemical Engineering, Indian Institute of Technology Kharagpur, Kharagpur, IND; 4 Nanotechnology, Indian Institute of Technology Roorkee, Roorkee, IND; 5 Dentistry, All India Institute of Medical Sciences, Kalyani, Basantpur, IND; 6 Oral and Maxillofacial Surgery, Dr. Harvansh Singh Judge Institute of Dental Sciences and Hospital, Chandigarh, IND

**Keywords:** carbon nanotubes, cytotoxicity, dental restorative materials, epoxy composites, graphene oxide, mechanical properties

## Abstract

Background

The increasing demand for mercury-free restorative materials has encouraged the development of alternative biomaterials with improved mechanical strength and biocompatibility.

Aim

To compare the mechanical and biological performance of carbon nanotube (CNT)- and graphene oxide (GO)-reinforced epoxy composites as potential restorative materials.

Objectives

To fabricate epoxy nanocomposites with varying CNT (1%, 2.5%, 5%) and GO (5%, 10%, 15%) concentrations and evaluate their tensile, compressive, nano-mechanical, and cytotoxic characteristics.

Materials and methods

Epoxy composites were synthesized using epoxy resin and hardener systems incorporated with CNT and GO nanofillers. Mechanical properties were evaluated through tensile, compressive, and nanoindentation testing, while cytotoxicity on human gingival fibroblasts was assessed using the crystal violet assay.

Results

GO-reinforced composites showed higher modulus and tensile strength than CNT-reinforced systems in this limited sample (n=2 per group). The apparent optimum reinforcement occurred at 10% GO and 2.5% CNT.

Conclusion

Controlled incorporation of CNTs and GO improved the mechanical performance of epoxy composites and was not associated with marked short-term cytotoxicity at lower loadings in a preliminary screening assay.

## Introduction

The gradual phase-down of dental amalgam following the Minamata Convention on Mercury has encouraged the search for mercury-free restorative materials [1]. Although several alternatives, including composite resins and glass ionomer cements, are available, none of them yet replicate amalgam’s unique combination of strength, durability, cost-effectiveness, and ease of manipulation [2,3]. This study aims to understand the fundamental mechanical behavior and cytotoxicity of epoxy-based nanocomposites, which may serve as a foundation for future dental applications. Epoxy resins demonstrate superior adhesion, minimal polymerization shrinkage, and excellent chemical stability [4]. However, reinforcement is essential to enhance their mechanical properties for clinical application [5].

Carbon-based nanomaterials, such as carbon nanotubes (CNTs) and graphene oxide (GO), have demonstrated exceptional reinforcing capabilities owing to their high surface area and intrinsic strength [6]. Yet, limited evidence exists regarding their performance within epoxy matrices relevant to dental applications [7,8]. Most previous dental nanocomposite studies have focused on silica or ceramic fillers, with almost none directly comparing CNT and GO in epoxy systems to determine which offers better reinforcement efficiency at lower loadings [9,10].

Investigation of CNT- and GO-reinforced epoxy composites at the laboratory level enables the identification of filler combinations and concentrations that achieve an optimal balance between strength and stability, thereby establishing a foundation for the development of next-generation mercury-free restorative materials [11]. Although numerous studies have investigated the reinforcement of epoxy matrices using either CNT or GO independently, direct comparative analyses under identical processing conditions remain limited. Furthermore, most existing investigations primarily focus on bulk mechanical properties, with comparatively fewer studies integrating nanoindentation-based local mechanical characterization with macroscopic tensile and compressive testing [12].

An epoxy matrix was used as a model system rather than a clinically established dental resin such as Bisphenol-A glycidyl methacrylate (Bis-GMA) or urethane dimethacrylate (UDMA)-based composites, since epoxy shares the same fundamental reinforcement mechanism - matrix-filler stress transfer governed by dispersion, aspect ratio, and interfacial adhesion that underlies filler-reinforced dental composites generally. Its low viscosity and room-temperature curing also allowed consistent filler dispersion across a wide concentration range, which is harder to achieve in photopolymerizable Bis-GMA/UDMA systems at high filler loadings. This work should therefore be regarded as a foundational, screening-level study to identify promising filler-concentration combinations, rather than a direct evaluation of a clinically deployable material. Translation into Bis-GMA/UDMA-based dental resins, followed by testing under clinically relevant conditions (light-curing, thermal/humidity cycling, wear), is a necessary next step for future work.

The present study aims to provide a systematic comparative evaluation of CNT- and GO-reinforced epoxy composites across varying filler concentrations using a multiscale mechanical approach. By correlating nano-scale mechanical properties with bulk performance and fracture morphology, this work elucidates the concentration-dependent reinforcement mechanisms of one-dimensional (CNT) and two-dimensional (GO) nanofillers within the same polymer system. This integrated framework offers clearer insight into filler geometry-property relationships and optimal reinforcement windows for restorative material applications.

Thus, the objectives of this study were: to fabricate epoxy composites incorporating varying concentrations of CNT (1%, 2.5%, and 5%) and GO (5%, 10%, and 15%); to evaluate their mechanical performance using tensile, compressive, and nanoindentation testing; and to compare the reinforcement efficiency to determine the most effective filler type and concentration. Baseline mechanical data for CNT- and GO-reinforced epoxy systems were established through a combination of macro-scale (universal testing machine, UTM) and micro-scale (nanoindentation) assessments [13]. This dual-scale testing approach provided an integrated understanding of how each nanofiller influences both the overall and localized mechanical behavior of the epoxy matrix, forming the groundwork for future research on mercury-free dental restorative systems. Cytotoxicity evaluation was performed using the crystal violet staining method to identify the composite formulation exhibiting the lowest toxicity and highest biocompatibility for potential clinical applications [14].

## Materials and methods

The epoxy matrix was prepared using Huntsman Araldite GY 250 epoxy resin and Aradur 951 hardener (Huntsman LLC, Mumbai, India). The two different nanofillers were investigated separately as reinforcing agents: research-grade multi-walled carbon nanotubes (the specific CNT type used in this study) supplied by AdNano Technologies Pvt. Ltd., and GO supplied by Shilpent (Shilpa Enterprises, Bangalore, India). CNTs and GO were incorporated independently into the epoxy matrix at designated concentrations to evaluate and compare their effects on the mechanical properties of the resulting nanocomposites. Elegoo Standard Grey Photopolymer Resin (3D Master India, Pune, India) was obtained for the fabrication of the 3D-printed master moulds. TINFLIX TLS-215 Liquid Silicone (Tinflix Techno Products Pvt Ltd., Bangalore, India) was obtained for the preparation of the flexible silicone casting moulds. All materials were procured from their respective manufacturers/suppliers and used as received without any further purification.

Design and fabrication of mould

**Figure 1:**
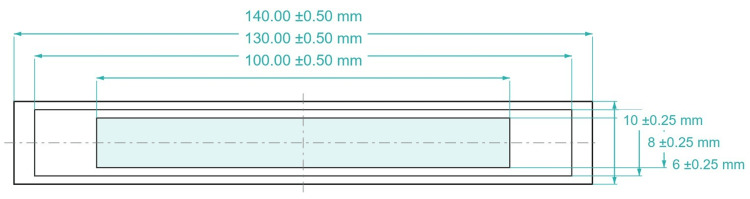
Rectangular mould for tensile strength testing Image credit: Created by the authors using OriginPro 2026b (64-bit) Beta2 10.3.5.62 software (⁠OriginLab Corporation, MA, US).

SolidWorks 2024 (Dassault Systèmes SolidWorks Corporation, Waltham, MA, USA), a computer-aided design (CAD) software package employed for creating the 3D mould geometries used in specimen fabrication software, was used to make the main designs for the moulds. The design included three different mould shapes, each of which was needed for a different kind of mechanical testing. A rectangular cuboid mould with an outer wall of 140 mm × 10 mm and an interior wall of 130 mm × 8 mm was made for tensile testing. The core part of the mould was 100 mm × 6 mm and 6 mm tall, which produced samples that could be tested for tensile strength using a UTM (Figure 1).

**Figure 2:**
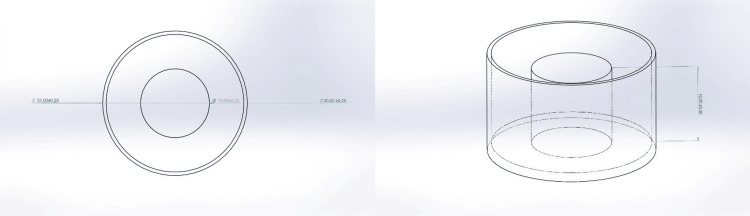
Cylindrical mould for compressive stress testing (ASTM D695) Image credit: Created by the authors using OriginPro 2026b (64-bit) Beta2 10.3.5.62 software (⁠OriginLab Corporation, MA, US).

A cylindrical mould with an outside wall diameter of 22 mm and an interior wall diameter of 21 mm was made for compressive testing. The primary cylinder was 12.7 mm in diameter and 25.4 mm in height, and it was meant for compressive UTM testing (Figure 2).

**Figure 3:**
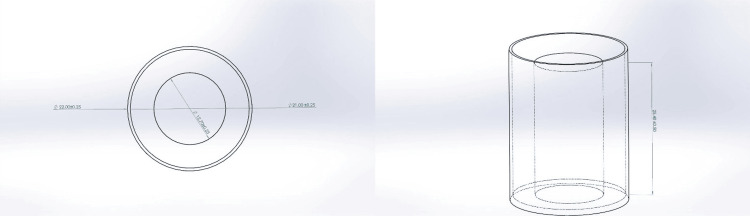
Cylindrical mould for nanoindentation testing (ASTM E2546) Image credit: Created by the authors using OriginPro 2026b (64-bit) Beta2 10.3.5.62 software (⁠OriginLab Corporation, MA, US).

A third, smaller cylindrical mould was also made for nano-indentation testing. It had an outside wall diameter of 21 mm and an inner wall diameter of 20 mm. The centre cylinder was extruded to a height of 10 mm and had a diameter of 10 mm (Figure 3).

Elegoo Standard Grey Photopolymer Resin and Elegoo Saturn 4 3D printer (both from 3D Master India, Pune, India) were used to make all the planned moulds. After that, liquid silicone rubber (LSR) was made by combining the silicone and binder in a 10:1 weight ratio. The mixture was then poured into the printed moulds to make the final flexible silicone moulds that were used for casting the samples.

Synthesis of GO and CNT-reinforced epoxy composites

The epoxy nanocomposites were fabricated using Araldite GY 250 epoxy resin and Aradur 951 hardener (Huntsman LLC, Mumbai, India) in accordance with the manufacturer's technical data sheet (TDS), which recommends a resin-to-hardener ratio of 10:1 by weight. Predetermined quantities of CNT (1 wt%, 2.5 wt%, and 5 wt%) or GO (5 wt%, 10 wt%, and 15 wt%) were gradually added to the epoxy resin and manually mixed using a glass stirring rod until a visually homogeneous dispersion was obtained. Subsequently, the hardener was incorporated and the mixture was further stirred to ensure uniform distribution of the nanofillers within the epoxy matrix. As the manufacturer's TDS specifies a pot life of approximately 25-40 minutes at room temperature, all formulations were processed immediately after mixing. The resulting mixtures were subjected to vacuum desiccation for approximately 10-15 minutes to remove entrapped air bubbles and minimize void formation within the cured composites. Following degassing, the mixtures were carefully poured into pre-fabricated silicone moulds corresponding to the required specimen geometries and allowed to cure at room temperature (25 ± 2 °C) for six hours. After curing, the specimens were demoulded and visually inspected for dimensional accuracy and curing completeness before mechanical characterization. Throughout the fabrication process, the recommended resin-to-hardener ratio and curing conditions were strictly maintained. However, one specimen from the 5 wt% GO group exhibited incomplete curing during compressive testing and was therefore excluded from the final analysis due to its unreliable mechanical response.

Characterization

The mechanical characterization of the fabricated nanocomposite samples was performed to assess their tensile, compressive, and nano-mechanical properties. All samples underwent preparation and testing following established procedures to guarantee the consistency and reliability of the results.

For each group, two samples were tested for tensile strength, two for compressive strength, and two for nanoindentation analysis. Additionally, two samples from each group were used for cytotoxicity evaluation. The mean values obtained from the tested samples were calculated and reported in the results section to ensure consistency and reliability of data.

This sample size (n=2 per group) was adopted as an initial screening approach to evaluate the feasibility and comparative behavior of CNT- and GO-reinforced epoxy composites across multiple filler concentrations and testing modalities. However, this limited replication restricts the statistical power of the study and precludes robust inferential analysis (e.g., variance estimation, hypothesis testing). Accordingly, all mechanical and cytotoxicity results in this study are presented as descriptive/preliminary trends rather than statistically validated differences between groups, and larger sample sizes are recommended in future work to confirm these findings.

Tensile and compressive testing

A Tinius Olsen H50KS UTM was used to perform tensile and compressive testing. For tensile testing, samples made from rectangular silicone moulds were loaded at a crosshead speed of 10 mm/minute. For compressive testing, cylindrical samples were loaded at a crosshead speed of 5 mm/minute. The experiments were performed at room temperature in a typical laboratory setting. The load-displacement data were captured in real time and utilized to understand the behavior of each composite sample under externally applied tensile/compressive stress.

Nano-indentation testing

A Hysitron TI 950 TriboIndenter (Bruker Corp., Massachusetts, US) was used to perform nano-mechanical characterization. All measurements were made using a diamond Berkovich indenter (Synton-MDP Ltd., Switzerland) that had a tip radius of 100 nm and a tip angle of 142.3°. The highest force applied was 4000 µN, and each indentation cycle included a 20-second loading, a 10-second hold, and a 20-second unloading. The resulting load-displacement curves were analyzed to determine the hardness and elastic modulus of the nanocomposite surfaces.

Surface morphology and elemental analysis

JEOL JSM-7610F Field Emission Scanning Electron Microscopy (FESEM) was used to analyze the surface morphology and dispersion of the nanofillers in the epoxy matrix. The elemental composition and the presence and distribution of carbon-based nanofillers (GO and CNT) in the cured composites were further confirmed by Energy Dispersive X-ray Spectroscopy (EDS) analysis. The samples were sputter-coated with a thin layer of gold before imaging in order to improve conductivity and avoid surface charge accumulation while being observed.

Cytotoxicity testing 

Cytotoxicity of the epoxy-based nanocomposites was evaluated using the crystal violet staining method on human gingival fibroblast (HGF) cells. The cells were cultured in Dulbecco’s Modified Eagle Medium (DMEM) supplemented with 10% fetal bovine serum and 1% penicillin-streptomycin under standard conditions (37 °C, 5% CO₂). After reaching 80-90% confluence, the cells were seeded into 96-well plates at a density of approximately 1×10⁴ cells/well and allowed to adhere for 24 h.

Extracts of the epoxy composites containing varying concentrations of CNT (1%, 2.5%, and 5%) and GO (5%, 10%, and 15%) were prepared by immersing sterilized samples in culture medium at a surface area-to-volume ratio of 3 cm²/mL and incubating for 24 h. The cells were then exposed to these extracts for another 24 h. Following exposure, the wells were washed with phosphate-buffered saline (PBS), fixed with 4% paraformaldehyde for 10 min, and stained with 0.5% crystal violet solution for 30 min at room temperature. Excess stain was removed by rinsing gently with distilled water, and the bound dye was solubilized using 33% acetic acid. The absorbance was measured at 570 nm using a microplate reader. Cell viability was expressed as a percentage relative to untreated control cells.

Statistical analysis

Given the small sample size (n=2 per group), formal inferential statistics (e.g., ANOVA, t-tests) were not performed, as such small samples do not permit reliable estimation of variance or meaningful p-values. Data are reported as mean ± range/SD where available (see nanoindentation data), and results are interpreted as descriptive trends rather than statistically validated differences between groups.

## Results

All mechanical results reported below (nanoindentation, tensile, and compressive) are derived from two specimens per group; given this limited replication, the values and comparisons presented should be interpreted as preliminary trends rather than statistically confirmed differences between filler types or concentrations.

Nanoindentation & UTM

**Figure 4:**
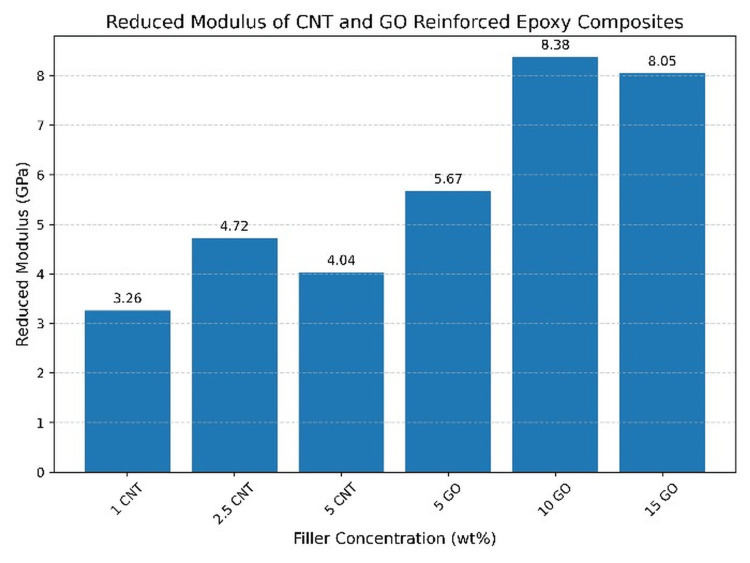
Reduced modulus of CNT- and GO-reinforced epoxy composites at varying filler concentrations CNT: carbon nanotube; GO: graphene oxide.

For CNT-reinforced composites, the reduced modulus increased from 3.26 GPa at 1% CNT to 4.725 GPa at 2.5% CNT, followed by a slight reduction to 4.035 GPa at 5% CNT (Figure 4).

Based on measurements obtained from two specimens per group (n=2), the mean reduced modulus increased from 3.26 GPa at 1 wt% CNT to 4.73 GPa at 2.5 wt% CNT. Given the limited sample size, these observations should be interpreted as preliminary trends rather than statistically validated differences.

This indicates that moderate CNT loading enhances stiffness through improved stress transfer and effective filler-matrix interaction. This substantial improvement in stiffness with increasing GO content can be attributed to the high aspect ratio and strong interfacial interaction of GO nanosheets with the epoxy matrix. The reduction at higher loading may be attributed to partial nanotube agglomeration, which disrupts uniform stress distribution within the matrix (Table 1).

**Table 1:** Nanoindentation results showing reduced modulus and hardness of CNT- and GO-reinforced epoxy composites at varying filler concentrations CNT: carbon nanotube; GO: graphene oxide.

Sample	Group	Reduced modulus (GPa)	Hardness (MPa)
CNT	1 CNT	3.26	209.5
	2.5 CNT	4.725	130
	5 CNT	4.035	217
GO	5 GO	5.675	225
	10 GO	8.38	140
	15 GO	8.05	185

**Figure 5:**
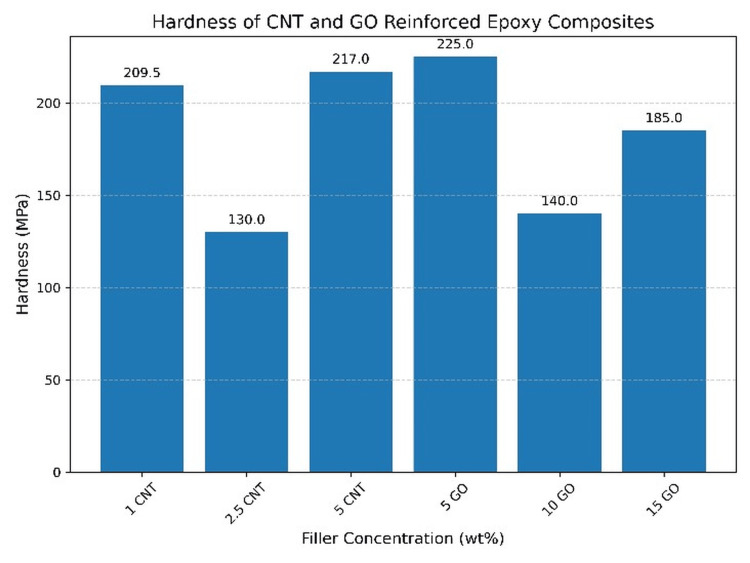
UTM results showing the tensile and compressive strength of CNT- and GO-reinforced epoxy composites at varying filler concentrations UTM: Universal testing machine; CNT: carbon nanotube; GO: graphene oxide.

The hardness behaviour of CNT composites demonstrated a non-linear trend. Hardness decreased from 209.5 MPa at 1% CNT to 130 MPa at 2.5% CNT, followed by an increase to 217 MPa at 5% CNT (Figure 5).

This U-shaped response suggests that intermediate loading may have resulted in localized dispersion heterogeneity, whereas higher loading potentially facilitated partial formation of an interconnected nanotube network, improving resistance to localized indentation. However, due to limited specimen number, this interpretation remains cautious.

Similarly, GO-reinforced composites exhibited an increase in reduced modulus from 5.675 GPa (5% GO) to a peak of 8.38 GPa (10% GO), followed by a slight decrease to 8.05 GPa (15% GO) (Figure 4). This trend suggests that optimal reinforcement occurs at moderate GO loading, beyond which nanosheet restacking and agglomeration may reduce effective stress transfer.

The hardness values for GO composites also showed concentration-dependent behaviour. Hardness decreased at 10% GO (140 MPa) compared to 5% GO (225 MPa), but increased again at 15% GO (185 MPa) (Figure 5). Such fluctuations may be associated with localized stress concentration, interfacial debonding, or nanosheet clustering affecting nano-scale indentation response.

**Figure 6:**
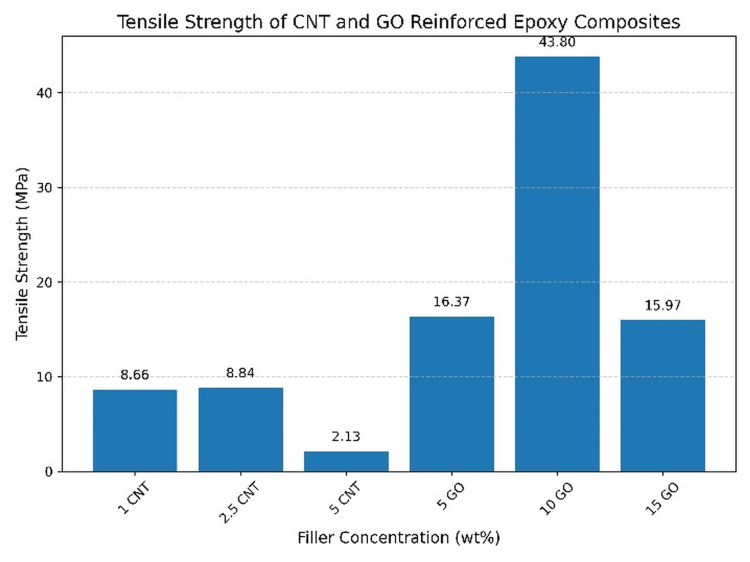
Tensile strength of CNT- and GO-reinforced epoxy composites at varying filler concentrations CNT: carbon nanotube; GO: graphene oxide.

Tensile testing revealed that CNT-reinforced composites achieved peak tensile strength at 2.5% CNT (8.835 MPa), followed by a substantial decline at 5% CNT (2.13 MPa) (Figure 6).

The reduction at higher loading likely reflects agglomeration-induced stress concentration and matrix discontinuity.

In contrast, GO-reinforced composites exhibited marked tensile enhancement at 10% GO (43.8 MPa), with reduced performance at 15% GO (Figure 6). This behaviour indicates that controlled GO dispersion at moderate loading improves crack deflection and interfacial adhesion, whereas excessive loading compromises matrix integrity.

Compressive strength results demonstrated improved load-bearing capacity at 2.5% CNT (337 MPa) and stable performance across 10% and 15% GO groups. One specimen in the 5% GO group exhibited improper curing and was excluded from interpretation, as incomplete polymerization resulted in surface softness and unreliable mechanical response. This observation underscores the sensitivity of nanocomposite performance to curing control and processing parameters.

Overall, both nanofillers enhanced mechanical properties in a concentration-dependent manner, with optimal performance observed at 2.5% CNT and 10% GO.

SEM analysis

**Figure 7:**
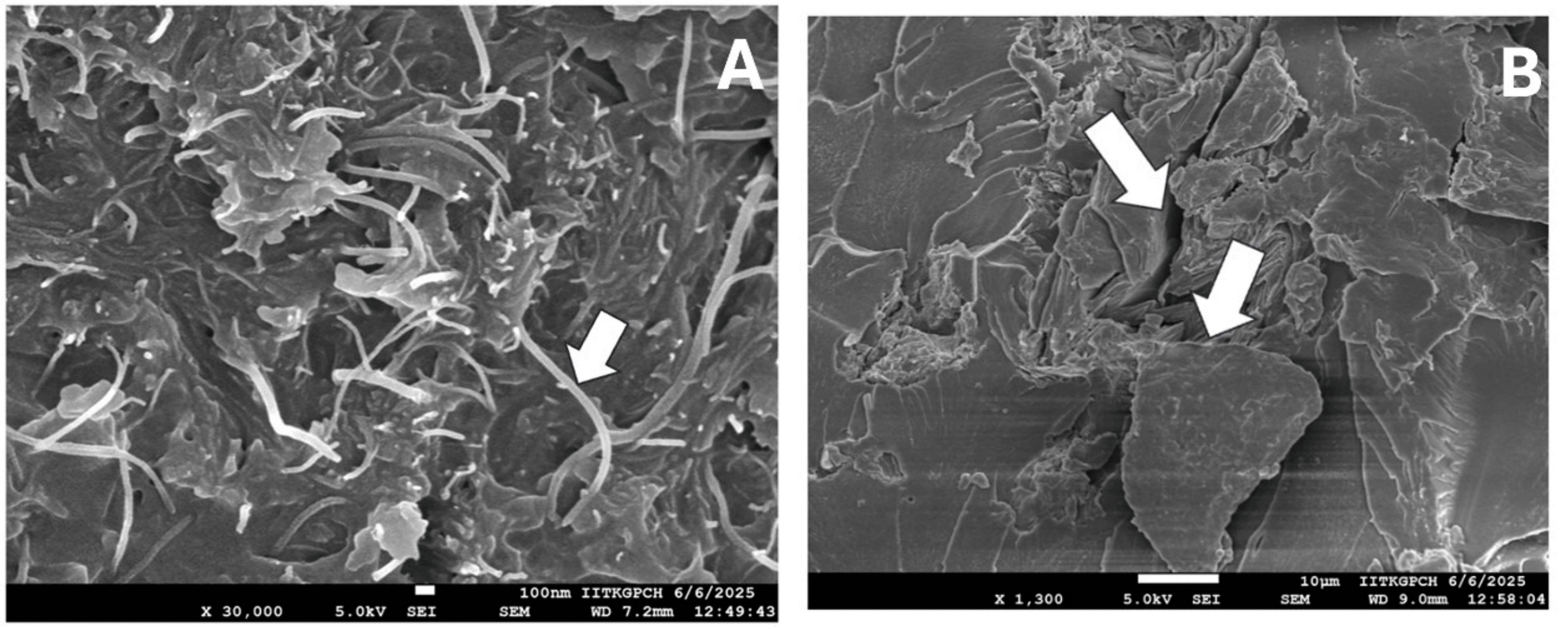
Scanning Electron Microscope (SEM) images CNT and GO CNT-reinforced composite showing nanotube pull-out, crack bridging, and clustering (30,000×); GO-reinforced composite showing lamellar nanosheets, sheet delamination, and localized agglomeration (1,300×). CNT: carbon nanotube; GO: graphene oxide.

The SEM micrograph of the CNT-reinforced composite (Figure 7a) shows elongated filamentous structures embedded within and protruding from the epoxy matrix, corresponding to nanotube pull-out during fracture.

The presence of partially extracted nanotubes indicates interfacial load transfer prior to failure. The fracture surface appears rough and irregular, suggesting localized energy dissipation during crack propagation. Regions of nanotube overlap and entanglement are visible, although large-scale agglomeration is not prominent (Figure 7).

In the GO-reinforced composite (Figure 7b), the fracture surface exhibits plate-like and lamellar features characteristic of graphene oxide nanosheets. Large fractured flakes and step-like cleavage patterns are observed, indicating crack propagation along interfaces influenced by rigid sheet inclusions. The fracture morphology appears comparatively more planar and layered than the CNT system, consistent with sheet-reinforced brittle fracture behaviour.

**Figure 8:**
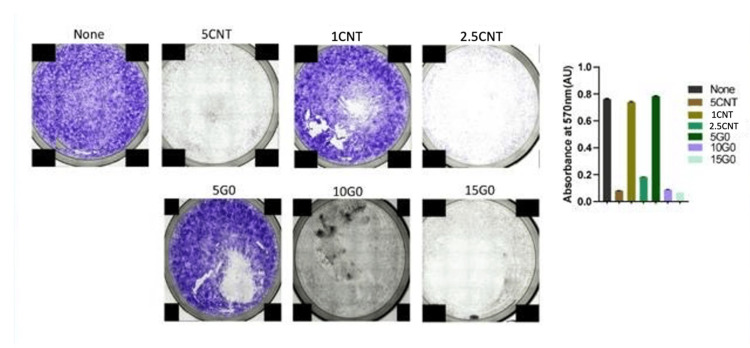
Crystal violet staining assay of human gingival fibroblast (HGF) cells exposed to epoxy–based nanocomposite extracts containing varying CNT and GO concentrations CNT (1%, 2.5%, and 5%) and GO (5%, 10%, and 15%) concentrations. Darker violet coloration indicates higher cell viability. CNT: carbon nanotube; GO: graphene oxide.

Overall, the CNT composite demonstrates a rougher fracture morphology with visible nanotube pull-out, whereas the GO composite exhibits layered fracture surfaces associated with planar nanosheet reinforcement (Figure 8).

Cytotoxicity

The crystal violet assay results (Figure 8) demonstrated concentration-dependent cytotoxicity for both CNT- and GO-reinforced epoxy composites. The control and 1% CNT, 5% GO groups retained dense violet staining, indicating high cell viability comparable to untreated fibroblasts. In contrast, 2.5% CNT and 5% CNT groups exhibited a marked reduction in staining intensity, reflecting decreased viable cell density. Among the GO-reinforced groups, 10% GO and 15% GO showed minimal or no staining, suggesting apparent trend towards cytotoxic effects at higher concentrations. Quantitative absorbance analysis at 570 nm confirmed these visual findings, with the 1% CNT group, 5% GO showing the highest viability among all reinforced composites. These observations indicate that lower CNT and GO concentrations exhibit better cytocompatibility, whereas higher CNT and GO loadings adversely affect cell proliferation due to increased surface reactivity and potential oxidative stress. It should be noted that these findings are based on a single 24-hour crystal violet staining assay, which serves as an indirect proxy for cell viability via relative cell density. They have not been confirmed using orthogonal methods such as live/dead fluorescence staining or metabolic activity assays, and should therefore be interpreted as preliminary screening data rather than definitive evidence of biocompatibility.

## Discussion

The incorporation of carbon-based nanofillers in polymeric matrices for dental applications has been shown to enhance mechanical performance through improved stress transfer, interfacial bonding, and filler dispersion [15-17].

Our observation that low concentrations of CNTs improve mechanical properties while GO requires higher, optimized loadings to reach peak performance and may exhibit deterioration at increased GO concentration is consistent with the existing literature. A systematic review by Sahm et al. (2023) discussed that graphene derivatives like GO, rGO, and graphene nanoplatelets improve the flexural strength, hardness, and modulus in dental polymers when added at low-to-moderate concentrations; however, benefits plateau or reverse when loadings exceed the optimal range owing to agglomeration and matrix disruption [18].

The concentration-dependent behavior observed in both CNT and GO systems reflects the critical role of filler dispersion and interfacial bonding. Multi-walled carbon nanotubes (MWCNTs) possess high aspect ratios (typically >100-1000), enabling effective stress transfer even at low loading levels [19]. At moderate concentrations, improved mechanical response may be associated with enhanced filler-matrix interaction and partial formation of an interconnected nanotube framework. However, excessive loading promotes agglomeration, reducing reinforcement efficiency. GO nanosheets, owing to their planar morphology and oxygen-containing functional groups, require optimized loading to maximize interfacial adhesion. Beyond the optimal concentration, restacking and increased resin viscosity may lead to matrix discontinuities and stress concentration zones, explaining the reduced performance at 15% GO.

Sharafeddin et al. (2023) found that incorporating 2 wt% GO into a resin-modified glass ionomer increased flexural strength, supporting the present study's finding that moderate GO loadings can markedly enhance bulk strength. Similarly, in dental polymer systems like polymethylmethacrylate (PMMA), modest additions of graphene-based fillers produced significant improvement in mechanical properties like microhardness, flexural strength, and elastic modulus [20,21]. GO improves polymer stiffness and hardness because its atomically thin, 2D lamellar geometry and oxygenated surface groups promote strong interfacial interactions and load transfer with polymer matrices when well-dispersed [22,23]. However, at higher loadings, GO tends to restack or agglomerate, markedly increasing resin viscosity. Both phenomena reduce effective interfacial contact, hinder stress transfer, and can introduce voids or weak interfaces that act as stress concentrators, which explains performance drops beyond an optimal filler concentration [24].

Similarly, existing literature regarding CNT in dentistry likewise supports effective reinforcement at low concentration. Experimental work on CNT-reinforced glass ionomer reportedly improved compressive strength and hardness at minimal CNT loading, consistent with our observation that CNTs gave efficient reinforcement at 1-2.5 wt% [25]. As MWCNTs have a very high aspect ratio and can form an effective load-bearing network at low concentrations; when well dispersed, they act as crack-bridging and stress-transfer elements, producing marked strength increment without large viscosity increase or cure inhibition [25].

At the same time, some studies reported either negligible improvement or reduced mechanical performance by inclusion of graphene fillers in PMMA resins. These contradictory findings may be attributed to factors such as the use of only commercially available materials with undisclosed graphene concentrations, patented formulations, and unspecified incorporation methods. Moreover, the likely minimal graphene content, maintained to preserve the aesthetic quality of the resin, could have limited its reinforcing effect. Therefore, the results of such studies cannot be considered fully reliable or directly comparable [26,27].

Studies show that cytotoxicity often increases with filler concentration and depends on surface chemistry and the degree of particle leaching; low loadings with good matrix encapsulation frequently present acceptable biocompatibility. Our observation that lower CNT and GO concentrations exhibited relatively higher biocompatibility aligns with previous reports emphasizing minimal effective filler loadings to achieve an optimal balance between mechanical performance and biological safety [28,29].

Limitations

The study was limited to laboratory-scale testing and short-term cytotoxic evaluation under static conditions. Factors such as thermal cycling, long-term durability, and in-vivo biocompatibility were not assessed, which may influence clinical performance.Furthermore, as an epoxy-based model system rather than a clinically used Bis-GMA/UDMA dental resin, direct clinical extrapolation of these findings is not yet warranted; validation in an actual dental restorative resin matrix is required before clinical translation can be considered.The study was limited to laboratory-scale testing and short-term cytotoxic evaluation under static conditions. Factors such as thermal cycling, long-term durability, and in-vivo biocompatibility were not assessed, which may influence clinical performance. Cytotoxicity was assessed using only a single 24-hour crystal violet staining assay, which provides an indirect measure of relative cell density rather than a direct readout of cell health or function; this assay alone is insufficient to establish biocompatibility. Future work should incorporate orthogonal viability assays such as live/dead fluorescence staining and metabolic activity assays (e.g., 3-(4.5- dimethylthiazol-2- yl)-2, 5-diphenyltetrazolium bromide assay (MTT) or resazurin/AlamarBlue), along with extended exposure durations (e.g., 48-72 hours or longer), to more robustly characterize the cytotoxic profile of these composites. Furthermore, as an epoxy-based model system rather than a clinically used Bis-GMA/UDMA dental resin, direct clinical extrapolation of these findings is not yet warranted; validation in an actual dental restorative resin matrix is required before clinical translation can be considered.

Future directions

Further studies should focus on optimizing nanofiller dispersion, performing long-term aging and fatigue analyses, and validating the composites in simulated oral environments. Hybrid or surface-functionalized nanofillers may also be explored to enhance interfacial bonding and reduce cytotoxic effects for improved clinical translation.

## Conclusions

Within the limitations of this preliminary in vitro study, several trend-level observations can be made regarding the performance of the modified epoxy dental composites. GO-reinforced systems showed the largest apparent gains in stiffness, hardness, tensile, and compressive properties, with the 10% GO concentration displaying the highest overall performance among the evaluated groups. Conversely, the CNT-reinforced composites appeared to achieve their optimal mechanical strength at lower concentrations, specifically around 2.5% CNT. However, because these observations are based on a restricted sample size (two per group), these apparent differences do not represent statistically validated conclusions and require confirmation with larger sample sizes. Ultimately, while the controlled incorporation of carbon-based nanofillers suggests an encouraging pathway toward improving mechanical behavior without marked short-term cytotoxicity at low loadings, further rigorous testing under dynamic clinical conditions is necessary.
